# Supplementary material for: Mechanisms governing the pioneering and redistribution capabilities of the non-classical pioneer PU.1
Source: Nat Commun. 2020 Jan 21;11:402. doi: 10.1038/s41467-019-13960-2 (PMC6972792; doi:10.1038/s41467-019-13960-2)
Supplement: Supplementary file 7 — Source data [file 41467_2019_13960_MOESM7_ESM.zip › Source_Data/Figure6/Figure6B_MotifScanOutput/ETS1_PU.1/homerResults/motif41.similar.html]

motif41

## Information for motif41

C
T
G
A
C
G
A
T
C
G
A
T
G
T
A
C
A
G
C
T
A
C
T
G
C
T
G
A
A
C
T
G
C
G
T
A
C
G
T
A
  
Reverse Opposite:  

A
C
G
T
A
C
G
T
A
G
T
C
A
G
C
T
T
G
A
C
C
T
G
A
A
C
T
G
C
G
T
A
C
G
T
A
G
A
C
T
  

|  |  |
| --- | --- |
| p-value: | 1e-33 |
| log p-value: | -7.739e+01 |
| Information Content per bp: | 1.811 |
| Number of Target Sequences with motif | 488.0 |
| Percentage of Target Sequences with motif | 3.08% |
| Number of Background Sequences with motif | 446.1 |
| Percentage of Background Sequences with motif | 1.39% |
| Average Position of motif in Targets | 100.1 +/- 51.7bp |
| Average Position of motif in Background | 98.2 +/- 69.2bp |
| Strand Bias (log2 ratio + to - strand density) | 0.0 |
| Multiplicity (# of sites on avg that occur together) | 1.02 |
| Motif File: | file (matrix) reverse opposite |

### Similar de novo motifs found

|  |  |  |  |  |  |  |  |
| --- | --- | --- | --- | --- | --- | --- | --- |
| Rank | Match Score | Redundant Motif | P-value | log P-value | % of Targets | % of Background | Motif file |
| 1 | 0.917 | C T G A G A C T C G A T A C G T G T A C C G A T A T G C G C T A A C T G C G T A C G T A | 1e-30 | -71.305997 | 1.81% | 0.62% | motif file (matrix) |
| 2 | 0.982 | A G C T A C G T A C G T A G T C A C G T T A C G C G T A A C T G C G T A C T G A C G T A G A C T T C A G | 1e-28 | -64.732715 | 1.78% | 0.65% | motif file (matrix) |
